# Identification of interleukin-11 as a comorbid risk factor for prostate cancer and Alzheimer’s disease using integrated bioinformatics and machine learning

**DOI:** 10.3389/fimmu.2026.1911726

**Published:** 2026-08-20

**Authors:** Mengxue Wang, Yanrong Qian, Enyao Huang, Chunyan Chu, Tian Gao, Shengrong Chen, Na Zhao, Caichen Luo, Yifan Liu, Xiejunhao Zheng, Haowen Hu, Bowen Han, Ming Chen, Weipu Mao, Wenchao Li

**Affiliations:** 1 School of Medicine, Southeast University, Nanjing, China; 2 Department of Pathology, Zhongda Hospital, Southeast University, Nanjing, China; 3 Department of Neurology, The Affiliated Suzhou Hospital of Nanjing Medical University, Suzhou Municipal Hospital, Gusu School of Nanjing Medical University, Suzhou, China; 4 Department of Urology, Zhongda Hospital, Southeast University, Nanjing, China

**Keywords:** Alzheimer’s disease, comorbid gene study, interleukin-11, machine learning, prostate cancer

## Abstract

**Background:**

Prostate cancer (PCa) and Alzheimer’s disease (AD) are age-related disorders with a complex epidemiological association and limited therapeutic options. Identifying shared molecular drivers may reveal new treatment targets.

**Objective:**

To identify common transcriptomic signatures between PCa and AD and validate the role of interleukin-11 (IL11) as a functional comorbidity factor.

**Methods:**

Multi-cohort transcriptomic datasets (GSE48350, GSE5281 and GSE28146 for AD; TCGA-PRAD, DKFZ2018 and MSKCC for PCa) were analyzed. Differential expression analysis and weighted gene co-expression network analysis (WGCNA) were performed, followed by a two-tier machine learning pipeline (Random Forest and LASSO Cox regression) to screen overlapping genes. Immune infiltration was evaluated by CIBERSORT and single-cell transcriptomics for PCa. The functional role of IL11 was assessed in RM-1 murine and DU145 human PCa cells using colony formation, wound healing, Transwell assays, and immunocompetent C57BL/6 orthotopic and subcutaneous xenograft models. The cognitive effects of IL11 were evaluated using Morris water maze (MWM) tests, and the neuropathological changes were assessed by detecting hippocampal amyloid-β (Aβ) deposition. Additionally, a cross-sectional analysis was performed in a population cohort (n=215) to investigate the associations between IL11 levels and AD pathological biomarkers.

**Results:**

A total of 455 shared candidate genes were identified, and a 10-gene signature (NDRG4, ISG15, IL11, ENO2, DYNC1I1, DNASE1, ATP6V1G2, ATCAY, ANLN, AGAP9) was established. The risk score effectively stratified PCa patients with poor progression-free interval (log-rank P < 0.001; AUC for 1-,3-,5-year = 0.78,0.73,0.70), validated in two external cohorts (DKFZ2018, MSKCC). IL11 was the top candidate and was significantly upregulated in both diseases. High IL11 expression correlated with an immunosuppressive microenvironment, characterized by increased M2 macrophages and regulatory T cells, and with higher tumor mutation burden. Single-cell analysis localized IL11 to a subset of cancer-associated fibroblasts. *In vitro*, IL11 treatment enhanced PCa cell proliferation, migration, and invasion. *In vivo*, intraperitoneal IL11 accelerated PCa tumor growth in mice. In the MWM test, IL11-treated mice exhibited significantly longer escape latencies and fewer platform crossings, which were accompanied by increased hippocampal Aβ deposition, suggesting that IL11 induced cognitive dysfunction. Cross-sectional cohort analyses further linked higher serum IL11 to reduced CSF Aβ42 and elevated p-tau181.

**Conclusion:**

IL11 acts as a shared risk factor related to PCa progression and cognitive decline in AD, representing a convergent molecular pathway and a potential therapeutic target for both age-related diseases.

## Introduction

Prostate cancer (PCa) and Alzheimer’s disease (AD) are both prototypical age-related disorders, and their incidence rises dramatically as populations age. Aging is the single most important risk factor for both diseases, and a growing body of evidence indicates that the aging process itself through mechanisms such as cellular senescence, chronic low-grade inflammation (inflammaging), mitochondrial dysfunction, etc., driving the pathophysiology of both PCa and AD (1–3). In PCa, patient genomics and mouse functional genetics have revealed that senescence acts as a barrier to metastatic progression, yet therapy-induced senescence (TIS) can paradoxically promote treatment resistance through the senescence-associated secretory phenotype (SASP) (4, 5). In AD, aging-related neuroinflammation, driven by inflammaging and peripheral immune dysregulation, has emerged as a central player in disease initiation and progression, shifting focus beyond traditional amyloid and tau pathology (6, 7). Despite therapeutic advances, both diseases are associated with substantial morbidity and mortality. PCa remains the most common cancer in men, with an estimated 313,000 new cases in the United States in 2025 alone, and metastatic disease remains incurable (5, 8). AD is the most common cause of dementia, affecting an estimated 57 million people worldwide in 2021, with prevalence expected to triple to 152.8 million by 2050 (3, 8, 9). The global costs of AD have reached approximately $1 trillion annually, while the economic burden of PCa care continues to escalate with aging populations (9, 10).

Epidemiological studies have consistently documented an inverse association between PCa and AD, with individuals diagnosed with cancer exhibiting a reduced risk of AD and vice versa (11). However, Mendelian randomization analyses have failed to establish bidirectional causality, suggesting that the observed association may be driven by shared biological processes rather than direct causal effects (11, 12). Moreover, a substantial body of evidence has revealed that androgen deprivation therapy (ADT), the cornerstone of advanced PCa treatment, is associated with a significantly elevated risk of AD, with some studies reporting up to a 40-50% higher risk (13). Mechanistically, ADT disrupts blood-brain barrier integrity and promotes peripheral immune cell infiltration into the brain, enhancing neuroinflammation and cognitive decline in preclinical AD models (9, 14). These findings underscore a complex, bidirectional relationship between PCa and AD, mediated by aging-associated inflammatory and senescent pathways. Therefore, an increasing number of scholars have been investigating the shared molecular mechanisms linking these two aging-related diseases to provide new insights into their common pathogenesis and to identify potential therapeutic targets that could benefit both patient populations.

Despite substantial research investments and technological advancements, both PCa and AD remain associated with significant unmet clinical needs. In PCa, although androgen deprivation therapy (ADT) and next-generation androgen receptor pathway inhibitors (ARPIs) are effective treatment for localized and advanced disease, 20~30% patients without radical surgery or radiotherapy still eventually progress to castration-resistant prostate cancer (CRPC), a stage characterized by invariably fatal disease relapse (15, 16). The emergence of therapy resistance driven by AR splice variants such as AR-V7, lineage plasticity, and tumor microenvironment-mediated immune evasion remain the primary clinical challenge, and objective response rates to immune checkpoint inhibitors in metastatic CRPC rarely exceed single digits (17, 18). The complexity of resistance mechanisms, combined with intratumoral heterogeneity, has hindered the development of durable, curative therapies for metastatic disease (16, 18). In AD, the therapeutic landscape is even more barren. Despite decades of research centered on the amyloid cascade hypothesis, truly disease-modifying therapies have remained elusive (19, 20). Recently approved anti-amyloid monoclonal antibodies, such as lecanemab and donanemab, have demonstrated modest cognitive benefit but are associated with significant adverse effects, including amyloid-related imaging abnormalities (ARIA), and their clinical meaningfulness remains debated (20–22). Existing symptomatic treatments, such as acetylcholinesterase inhibitors and memantine, provide only temporary, short-term relief without altering disease progression (19). Consequently, both PCa and AD are characterized by a critical lack of effective therapeutic targets that can meaningfully alter disease trajectories.

Given the aging global population and the increasing coexistence of these two diseases in elderly individuals, there is an urgent and unmet need to identify novel functional targets with clinical translational potential (23). Therefore, in the present study, through integrated analysis of multi-cohort transcriptomic datasets and systematic machine learning-based screening, we identified potential comorbidity genes for AD and PCa, with IL11 being the most prominent candidate. We further explored the function and pathogenic mechanisms of IL11 using single-cell transcriptome analysis and immune infiltration deconvolution algorithms. Finally, we validated the role of IL11 in the initiation and progression of AD and PCa through functional and behavioral data.

## Methods

### Data acquisition and preprocessing

To systematically explore shared transcriptomic signatures between AD and PCa, we retrospectively retrieved gene expression data from publicly available repositories. For AD, three RNA microarray datasets: GSE28146 (24), GSE48350 (25) and GSE5281 (26) were downloaded from the Gene Expression Omnibus (GEO) database (https://www.ncbi.nlm.nih.gov/geo/). GSE48350 includes postmortem hippocampal and entorhinal cortex samples from 61 AD patients and 55 non-demented controls. GSE5281 consists of 87 brain tissue samples from multiple regions (entorhinal cortex, hippocampus, superior frontal gyrus, and primary visual cortex) across 33 AD cases and 30 controls. GSE28146 contains 22 postmortem hippocampal samples from AD patients and 22 age-matched controls. To minimize batch effects across the three AD microarray datasets (GSE48350, GSE5281, and GSE28146), we applied the ComBat algorithm from the sva R package. Principal component analysis (PCA) was performed on both pre- and post-correction data to confirm effective removal of technical variations while retaining biological differences between disease and control groups.

For PCa, RNA-seq data (counts per million, CPM) and corresponding clinical information were obtained from The Cancer Genome Atlas (TCGA-PRAD) project via the Genomic Data Commons (https://portal.gdc.cancer.gov/). The dataset comprises 499 primary prostate tumor samples and 52 adjacent normal prostate tissue samples. For external validation of prognostic signatures, we collected five independent PCa cohorts: DKFZ2018 (German Cancer Research Center, n=321), MSKCC (Memorial Sloan Kettering Cancer Center, n=140), GSE116918 (n=257), GSE21034 (n=131), and GSE107299 (n=106). Raw expression data from GEO were normalized using the robust multi-array average (RMA) algorithm with background correction and quantile normalization. RNA-seq data were transformed with variance-stabilizing transformation (VST) implemented in the DESeq2 package. The “BEST” tool (https://rookieutopia.hiplot.com.cn/app_direct/BEST/) was used to facilitate the retrieval, expression standardization, and survival data integration across multiple PCa cohorts. All data processing steps were performed using R software (version 4.2.2, R Foundation for Statistical Computing).

### Differential expression analysis and weighted gene co-expression network analysis

Differential expression analysis was conducted separately for each AD microarray dataset using the “limma” package (version 3.54.2). Linear models were fit with empirical Bayes moderation, and multiple testing was corrected by the Benjamini-Hochberg method. For TCGA-PRAD RNA-seq data, the “DESeq2” package (version 1.38.3) was used with a negative binomial generalized linear model. Differentially expressed genes (DEGs) were defined by an adjusted P-value (false discovery rate, FDR) < 0.05 and an absolute log2 fold change (|log2FC|) > 0.5. For AD, only genes consistently differentially expressed in at least two of the three datasets were retained to reduce platform-specific noise.

Weighted gene co-expression network analysis (WGCNA, version 1.72-1) was performed separately on the AD and PCa datasets to identify gene modules associated with disease status. Samples were first clustered to detect outliers (hierarchical clustering with a cut height of 15 cm, based on the function “hclust”). The soft-thresholding power (β) was selected by evaluating scale-free topology fit indices (R² > 0.85) over a range from 1 to 20. For AD, the optimal β was 9; for PCa, β was 7. An adjacency matrix was constructed using the selected β, then transformed into a topological overlap matrix (TOM). Hierarchical clustering of the TOM-based dissimilarity (1-TOM) was performed, and modules were identified by dynamic tree cutting (minimum module size = 30, deepSplit = 2). Modules with similar expression profiles were merged at a cut height of 0.25. For each module, the module eigengene (first principal component) was calculated, and its Pearson correlation with disease trait (AD vs. control, or PCa tumor vs. normal) was computed. Modules showing the highest absolute correlation coefficients (|r| > 0.5 and P < 0.05) were selected for further analysis.

### Identification of AD-PCa comorbidity gene signatures

We systematically intersected gene sets derived from differential expression analysis and WGCNA. First, for AD and PCa separately, we identified up-regulated DEGs (log2FC > 0.5) and down-regulated DEGs (log2FC < −0.5). The overlapping up-regulated genes (AD-up and PCa-up) and down-regulated genes (AD-down and PCa-down) were obtained. Second, from WGCNA, positively correlated modules (with disease) and negatively correlated modules were extracted. The intersection of positively correlated module genes from AD and PCa, as well as the intersection of negatively correlated module genes, was calculated. Finally, the union of all four intersection sets (AD-up and PCa-up, AD-down and PCa-down, AD-pos and PCa-pos, AD-neg and PCa-neg) was taken as the candidate comorbidity gene pool, which contained 455 unique genes.

### Functional enrichment and immune landscape analysis

Functional enrichment analyses were performed using the “clusterProfiler” package (version 4.6.2). Gene Ontology (GO) terms (Biological Process, Cellular Component, Molecular Function), Kyoto Encyclopedia of Genes and Genomes (KEGG) pathways, and Reactome pathways were examined. Enrichment was considered significant when the FDR-adjusted P-value (Benjamini-Hochberg method) was < 0.05. A protein-protein interaction (PPI) network of the 455 candidate genes was constructed using the STRING database (version 11.5) with a combined interaction score threshold of 0.4. The network was visualized and analyzed in Cytoscape (version 3.9.1) to identify hub genes based on degree centrality and betweenness centrality.

The immune infiltration landscape was evaluated using the CIBERSORT algorithm (27) (with 1,000 permutations) to estimate the relative proportions of 22 infiltrating immune cell subtypes in both AD and PCa samples. For cross-validation, we also applied the ESTIMATE algorithm to calculate immune and stromal scores, the TIMER database to assess correlation between gene expression and immune cell abundance, the MCPcounter method to quantify ten immune and stromal cell populations, and the xCell algorithm for 64 immune cell types. Pearson correlation coefficients were calculated between expression levels of the candidate hub genes and the abundance of each immune cell type across all samples. All immune deconvolution analyses were conducted using default parameters as provided in the respective R packages or online tools.

### Construction and validation of the prognostic machine learning model

A multi-step machine learning pipeline was used to screen robust diagnostic and prognostic biomarkers. First, a Random Forest (RF) model was built using the “randomForestSRC” package (version 3.2.0) on the combined AD datasets (as a classification task: AD vs. control). The number of trees (ntree) was set to 1,000, and the number of variables sampled at each split (mtry) was tuned by grid search from √p to p/3 (p = number of genes). Gene importance was ranked by Mean Decrease Accuracy (MDA) and Mean Decrease Gini (MDG). Genes with an MDA > 0.01 and MDG greater than the 75th percentile across all genes were retained.

Next, these retained overlapping genes were subjected to Least Absolute Shrinkage and Selection Operator (LASSO) Cox regression using the “glmnet” package (version 4.1-7) on the TCGA-PRAD dataset. Ten-fold cross-validation was performed to select the optimal penalty parameter λ, which was chosen as λ.min (the λ giving minimum partial likelihood deviance). Genes with non-zero coefficients at λ.min were identified as candidate predictors.

A multivariate Cox proportional hazards model (function coxph in the “survival” package, version 3.5-5) was then fitted using the selected genes. The final prognostic risk score was calculated as:

Risk Score = Σ (Expression of Gene_i × Coefficient_i).

Patients in the TCGA-PRAD cohort were stratified into high-risk and low-risk groups according to the median risk score. The same formula and cutoff were applied to the five external validation cohorts. Kaplan-Meier (K-M) survival analysis with log-rank test was used to compare biochemical recurrence-free survival (BCR-FS) between risk groups. Time-dependent receiver operating characteristic (ROC) curves and the area under the curve (AUC) were computed using the “timeROC” package (version 0.4) at 1, 3, and 5 years to evaluate predictive accuracy. A nomogram integrating the risk score and clinical variables (T stage, Gleason score, PSA level) was constructed using the “rms” package (version 6.8-0) and calibrated with 1,000 bootstrap resamples to assess prediction accuracy.

### Morris water maze test

Recombinant mouse IL-11 (rmIL-11, TargetMol, TMPY-04178) was reconstituted in sterile PBS at a concentration of 5 μg/200 μL. C57BL/6 mice received daily i.p. injections of rmIL-11 at a dose of 0.5 μg per mouse or an equivalent volume of vehicle control (PBS) for 10 consecutive days. All injections were performed at the same time each day between 9:00 and 10:00 AM to minimize circadian variability. Body weight and general health status were monitored daily throughout the treatment period.

The MWM test was employed to evaluate spatial learning and memory. The apparatus consisted of a circular pool (120 cm in diameter, 40 cm in height) filled with water to a depth of 30 cm, maintained at 21 ± 1 °C. The pool was virtually divided into four equal quadrants. A transparent circular escape platform (10 cm in diameter) was placed 1.0 cm below the water surface in a fixed quadrant throughout the experiment. During the acquisition phase, mice underwent five consecutive days of training to locate the submerged platform, with each trial lasting up to 60 seconds. Each day, the animals were gently placed into the water from four pseudo-randomly selected starting points, facing the wall. If a mouse failed to find the platform within the allotted time, it was manually guided onto the platform and allowed to remain there for 30 seconds. Escape latency and swimming trajectory were recorded using a video tracking system. On the sixth day, a probe trial was conducted to assess memory retention, during which the platform was removed. Mice were allowed to swim freely for 60 seconds, and the time spent in the target quadrant as well as the number of platform site crossings were recorded.

### Immunofluorescence staining assay

IF of Aβ in mouse brain tissue. One day after the completion of the MWM test, we conducted tissue sampling on the mice. Paraffin-embedded brain sections were subjected to deparaffinization, rehydration, and antigen retrieval. After cooling to room temperature, sections were blocked with 3% bovine serum albumin (BSA) in PBS for 30 min at room temperature. Subsequently, sections were incubated overnight at 4 °C in a humidified chamber with a mouse anti-β-Amyloid antibody (clone 6E10, 1:200, BioLegend, Cat# 803001, RRID: AB_2564653) diluted in 1% BSA in PBS. After washing three times with PBST (0.1% Tween-20 in PBS) for 5 min each, the sections were incubated with an Alexa Fluor secondary antibody (Beyotime, Shanghai, China) for 2 h at room temperature, followed by a coverslipping with an Antifade Mounting Medium containing DAPI (Beyotime, Cat# P0131). All sections were observed under a confocal microscope (ZEISS, Germany) and analyzed using the ImageJ software.

IF of CD206 and FOXP3 in mouse orthotopic tumor. Orthotopic prostate tumor tissues were fixed in 4% paraformaldehyde, cryosectioned at 5–8 μm, and blocked with 5% normal serum. Sections were incubated overnight at 4 °C with primary antibodies against CD206 (Abcam ab64693, 1:200) and FOXP3 (eBioscience 17-5773-82, 1:100). After washing, sections were incubated with Alexa Fluor-conjugated secondary antibodies (1:500) for 1 h at room temperature, counterstained with DAPI, and mounted. Images were acquired using a confocal microscope, and positive cells were quantified in at least five random fields per section using ImageJ.

### Acquisition and analysis of ADNI cohort data

Participants with available serum IL-11 levels, cerebrospinal fluid (CSF) Aβ42, and p-tau181 data were extracted from the Alzheimer’s Disease Neuroimaging Initiative (ADNI) database (https://adni.loni.usc.edu/) (28). A total of 215 participants were finally included, comprising 98 cognitively normal controls, 59 individuals with mild cognitive impairment (MCI), and 58 patients with Alzheimer’s disease (AD). Serum IL-11 concentrations were measured using the Olink^®^ HT Explore proteomics assay panel (29). CSF Aβ42 levels were assessed with the Elecsys^®^ β-amyloid(1-42) assay (Roche Diagnostics) (30), and CSF p-tau181 levels were determined using the Roche Elecsys^®^ phosphorylated Tau(181P) CSF immunoassay (31). Cognitive function was evaluated by the Mini-Mental State Examination (MMSE), with higher scores indicating better cognitive performance.

### *In vitro* experimental validation

#### Cell culture

The human prostate cancer cell line DU145 (obtained from American Type Culture Collection, ATCC HTB-81) and the RM-1 murine prostate carcinoma cell line (ATCC CRL-3310) were maintained in Dulbecco’s Modified Eagle Medium (DMEM, high glucose, Gibco) supplemented with 10% fetal bovine serum (FBS, Gibco, 42F8354K) and 1% penicillin-streptomycin (100 U/mL and 100 μg/mL, respectively) in a humidified incubator at 37 °C with 5% CO_2_. Cells were passaged every 3–4 days and used between passages 5 and 15 for all experiments.

#### Generation of fluorescent protein-expressing RM-1 cells

RM-1 cells were transduced with a lentiviral vector encoding eGFP and a puromycin resistance gene at an MOI of 10–20 in the presence of polybrene (8 μg/mL). After 24 h, the medium was replaced, and stable transductants were selected with puromycin (2 μg/mL). eGFP expression was confirmed by fluorescence microscopy.

#### Wound healing assay

Cells were seeded into 6-well plates (5 × 10^5^ cells/well) and grown to 90-95% confluence. A straight scratch was made across each well using a sterile 200-μL pipette tip. After washing twice with phosphate-buffered saline (PBS) to remove detached cells, the medium was replaced with serum-free DMEM containing recombinant human interleukin-11 (rhIL-11, R&D Systems, 218-IL) at concentrations of 0 (control, vehicle PBS), 10 ng/mL, or 50 ng/mL. Phase-contrast images were captured at 0, 12, and 24 hours post-scratch using an inverted microscope (Olympus CKX53) with a calibrated reticle. Wound closure area was quantified in three randomly selected fields per well using ImageJ software (NIH, version 1.53t). Each experiment was performed in triplicate wells and repeated independently three times. The percentage of wound closure was calculated as (initial area-remaining area)/initial area × 100%.

#### Colony formation assay

Cells were trypsinized and seeded into 6-well plates at a density of 1 × 10³ cells per well in complete medium. After 24 hours for attachment, cells were treated with rhIL-11 (0, 10, 50 ng/mL) in fresh medium. Medium with or without IL-11 was replaced every 3 days. After 14 days, colonies were fixed with 4% paraformaldehyde (Electron Microscopy Sciences) for 15 minutes and stained with 0.1% crystal violet (Sigma-Aldrich) for 20 minutes at room temperature. Colonies containing > 50 cells were counted manually under a stereomicroscope. The experiment was conducted with three independent biological replicates, each with duplicate wells.

#### Transwell migration assay

Cell migration was evaluated using 24-well Transwell chambers with 8.0-μm pore size polycarbonate membranes (Corning, 3422). DU145 cells were starved in serum-free DMEM for 12 hours, then harvested and resuspended in serum-free DMEM at 5 × 10^4^ cells per 200 μL. The cell suspension was added to the upper chamber. The lower chamber was filled with 600 μL DMEM containing 10% FBS as a chemoattractant. rhIL-11 was added to both chambers at the indicated final concentrations (0, 10, 50 ng/mL). After incubation at 37 °C for 12 hours, non-migrated cells on the upper side of the membrane were removed gently with a cotton swab. Migrated cells on the lower side were fixed with 4% paraformaldehyde (15 min) and stained with 0.1% crystal violet (20 min). Stained cells were photographed in five random fields per membrane (200× magnification) and counted using ImageJ. Each condition was tested in triplicate chambers, and the entire experiment was repeated three times.

### *In vivo* tumor xenograft model

All animal procedures were approved by the Institutional Animal Care and Use Committee of Zhongda Hospital, Southeast University (2025ZDSYLL297-P01). Male C57BL/6 mice (6–8 weeks old, body weight 20–22 g) were obtained from the Jackson Laboratory (USA) and housed under specific pathogen-free conditions with a 12-hour light/dark cycle, controlled temperature (22 ± 2 °C), and humidity (50 ± 10%). Food and water were provided ad libitum.

The murine prostate cancer cell line RM-1 was cultured in DMEM medium supplemented with 10% FBS and 1% penicillin-streptomycin. For tumor inoculation, cells were harvested at 80-90% confluence, washed twice with ice-cold PBS, and resuspended in PBS at a concentration of 1 × 10^7^ cells/mL.

#### Orthotopic RM-1 PCa xenograft model

Male C57BL/6 mice (6–8 weeks) were anesthetized and a lower midline incision was made to expose the prostate. RM-1 cells (2.5 × 10^5^ in 50 μL PBS) were injected into the subcapsular area of each prostate lobe. The wound was closed in layers. For bioluminescence tracking, RM-1-Luc cells were used, and tumor growth was monitored weekly using an IVIS imaging system following D-luciferin injection (150 mg/kg, i.p.). At endpoint, tumors were excised, weighed, and processed for histology or molecular analyses.

#### Subcutaneous RM-1 PCa xenograft model

Each mouse received a subcutaneous injection of 100 μL cell suspension (1 × 10^6^ cells) into the right dorsal flank using a 27-gauge needle. Seven days after injection, when palpable tumors had formed (~50 mm³), mice were randomly assigned to two groups (n = 8 per group): (i) IL-11 treatment group, receiving recombinant mouse interleukin-11 (rmIL-11, TargetMol, TMPY-04178) at 10 mg/kg body weight via intraperitoneal (i.p.) injection twice weekly (every 3 days); and (ii) control group, receiving an equivalent volume of sterile normal saline (0.9% NaCl). The dosage and schedule were based on previous pharmacokinetic studies in mice (unpublished observations and pilot experiments). Tumor dimensions (length, L; width, W) were measured every two days with a digital caliper (Mitutoyo, accuracy 0.01 mm). Tumor volume was calculated using the formula V = L × W²/2. Body weights of mice were recorded at the same time points to monitor general health.

After three weeks of treatment, mice were euthanized by carbon dioxide inhalation followed by cervical dislocation. Tumors were carefully dissected, excised, and weighed. Fresh tumor tissues were snap-frozen in liquid nitrogen for subsequent molecular analyses or fixed in 4% paraformaldehyde for histology. No mice showed signs of severe distress (weight loss > 20%, lethargy, or inability to reach food/water) during the study, and all animals were included in the final analysis. Statistical comparisons between groups for tumor volume and weight were performed using two-way repeated-measures ANOVA followed by Bonferroni post-hoc test (tumor volume over time) or unpaired two-tailed Student’s t-test (final tumor weight), with P < 0.05 considered statistically significant.

### Statistical analysis

All statistical analyses were performed using R software (version 4.2.2). Continuous variables were compared using the Wilcoxon rank-sum test or Kruskal-Wallis test. Survival differences were analyzed using the log-rank test. For multiple Spearman’s correlation tests, P-values were corrected using the Holm-Bonferroni method to control the family-wise error rate. Only correlations with adjusted P < 0.05 were considered statistically significant.

## Results

### Identification of shared transcriptomic signatures between AD and PCa

**Figure 1:**
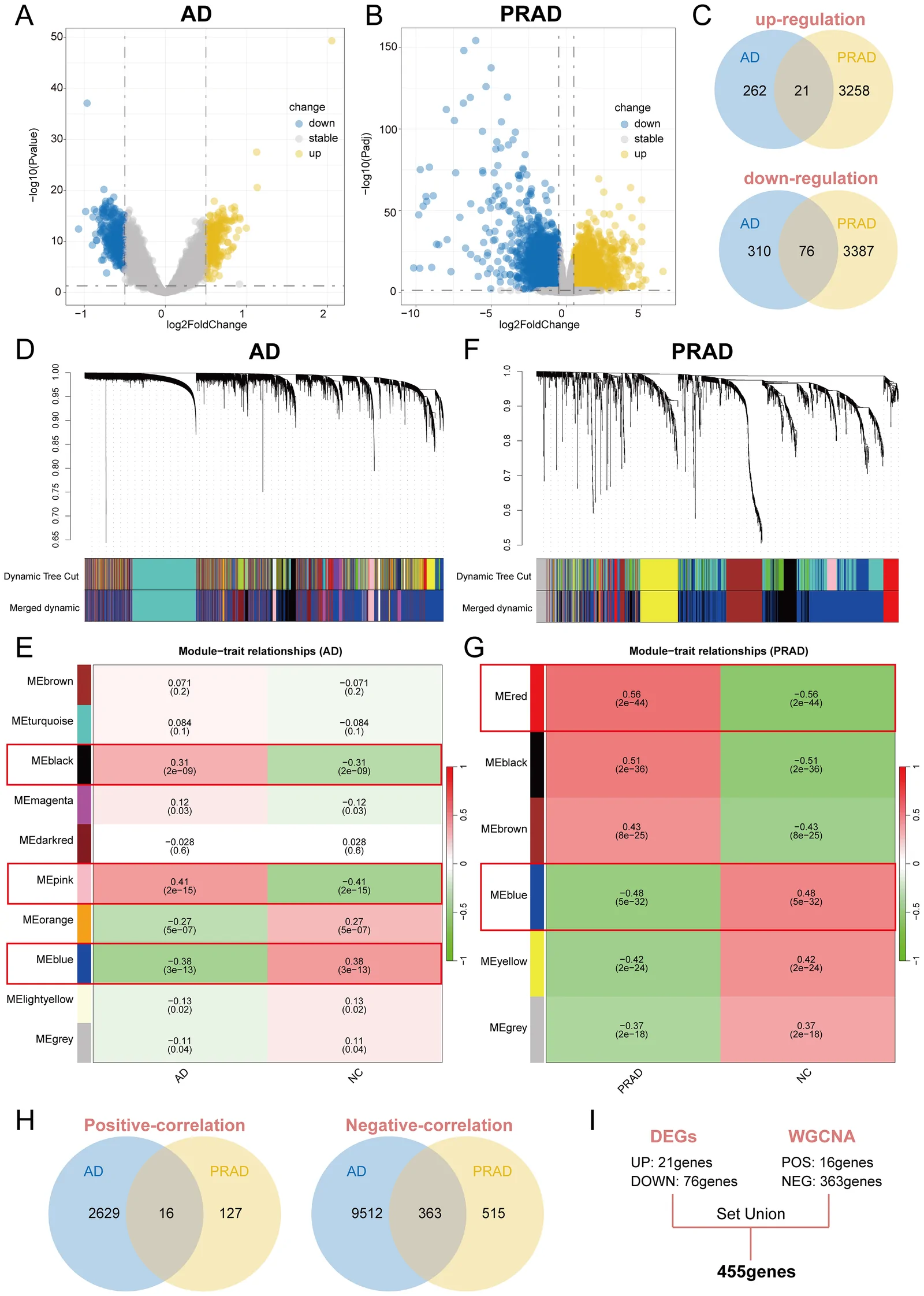
Identification of comorbidity genes between Alzheimer’s disease (AD) and prostate cancer (PCa) by integrating differential expression analysis and WGCNA. **(A)** Volcano plot showing differentially expressed genes (DEGs) (up-regulated in yellow, down-regulated in blue) in brain tissue RNA-seq data from AD patients compared to healthy controls, integrating GSE48350, GSE5281 and GSE28146 datasets. Genes with false discovery rate (FDR) < 0.05 and absolute log2 fold change (|log2FC|) > 0.5 are considered to be significant. **(B)** Volcano plot showing differentially expressed genes (DEGs) (up-regulated in yellow, down-regulated in blue) in tissue RNA-seq data of PCa samples compared to normal controls from TCGA-PRAD. Genes with false discovery rate (FDR) < 0.05 and absolute log2 fold change (|log2FC|) > 0.5 are considered to be significant. **(C)** Venn diagrams showing the commonly up-regulated (*top*) and down-regulated (*bottom*) genes in AD and PCa. **(D)** Waterfall plot illustrating the correlation between module eigengenes and disease traits of AD based on WGCNA. **(E)** Heatmap depicting the correlation between module eigengenes and clinical traits of AD identified by WGCNA. **(F)** Waterfall plot illustrating the correlation between module eigengenes and disease traits of PCa based on WGCNA. **(G)** Heatmap depicting the correlation between module eigengenes and clinical traits of PCa identified by WGCNA. **(H)** Venn diagram showing genes that are commonly positively or negatively correlated with disease traits of AD and PCa. **(I)** Union of DEGs and disease associated genes identified 455 comorbidity genes between AD and PCa.

To elucidate the molecular connection between AD and PCa, we first performed differential expression analyses at mRNA levels. To identify the differential genes between populations with and without AD, we first obtained and integrated three RNA-microarray datasets of patients from GEO database with open-access (GSE28164 (24), GSE48350 (25) and GSE5281 (26)). After the removal of batch effects and PCA assessment (**Supplementary Figures 1A, B**), the overall DEGs are shown through comparative analysis (**Figure 1A**). Similarly, a large number of significant differential genes in the TCGA-PRAD dataset were also identified through comparative analysis between the tumor group and the normal group (**Figure 1B**). These results indicated that both diseases exhibit widespread transcriptomic reprogramming relative to their respective controls. Intersection analysis revealed a core set of 21 up-regulated and 76 down-regulated genes shared between AD and PCa (**Figure 1C**). The presence of such common DEGs suggested that AD and PCa may share certain transcriptional alterations despite their distinct tissue origins.

To identify gene co-expression modules associated with disease status, we performed WGCNA with a Pearson correlation matrix and applied a soft-thresholding power of β = 4 for AD and β = 5 for PRAD to ensure scale-free topology (**Supplementary Figures 1C–H**). For module-trait correlation, a Pearson’s correlation coefficient threshold of |r| > 0.3 and a significance threshold of P < 0.05 were used to define modules that were significantly associated with the clinical traits of interest. Modules meeting these criteria were considered for downstream functional enrichment analyses. In AD, the “black” and “pink” modules showed strong positive correlations with disease status, while the “blue” module was negatively correlated (**Figures 1D, E**). In PCa, the “red” module was significantly positively associated with tumor status, and the “blue” module was negatively associated (**Figures 1F, G**). These module-trait relationships suggested that distinct co-expression networks are perturbed in each disease. By integrating the shared DEGs with genes from these disease-correlated WGCNA modules (**Figure 1H**), we established a consensus set of 455 candidate genes for further investigation (**Figure 1I**). This union approach ensured that genes identified by either differential expression or network-based methods were retained for downstream analysis.

### Functional profiling and immune landscape of the shared signature in AD and PCa

**Figure 2:**
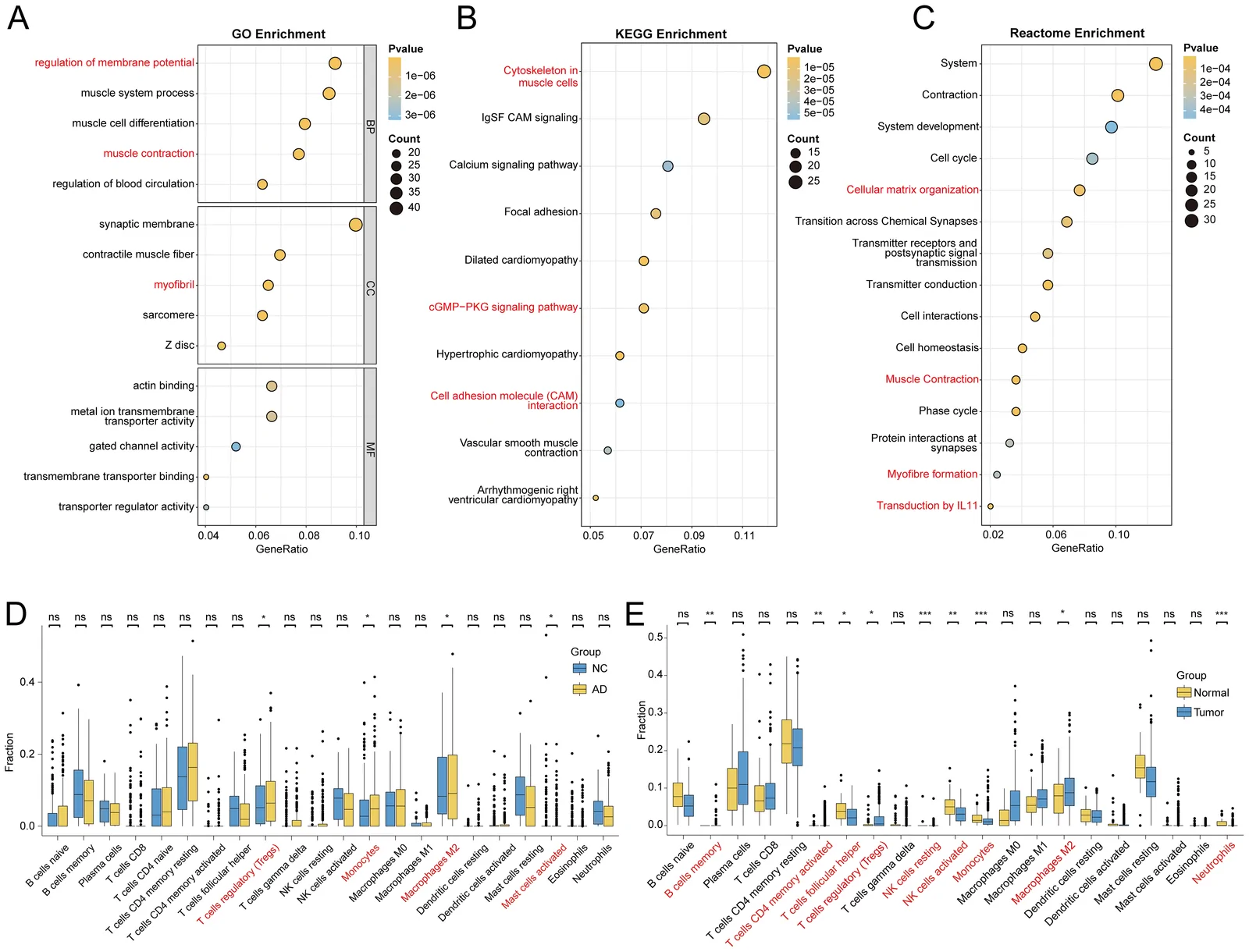
Biological functions and associated immune features of comorbidity genes between AD and PCa. **(A)** Bubble plot showing GO enriched biological signaling pathways of the 455 AD-PCa comorbidity genes. **(B)** Bubble plot showing KEGG enriched biological signaling pathways of the 455 AD-PCa comorbidity genes. **(C)** Bubble plot showing Reactome enriched biological signaling pathways of the 455 AD-PCa comorbidity genes. **(D)** Box plots comparing the differences in infiltration levels of 22 immune cell types between the AD group and the healthy control group. *p < 0.05, **p < 0.01, by two tailed unpaired t test. Multi-comparisons were corrected using False Discovery Rate (FDR). The boxes represent the median and interquartile range (IQR), with the whiskers representing the data range (1.5 × IQR). Immune cell infiltration levels were predicted by deconvolution using the CIBERSORT algorithm. Differential immune cells with significance were marked in red color. **(E)** Box plots comparing the differences in infiltration levels of 22 immune cell types between the PCa group and the normal group. *p < 0.05, **p < 0.01, ***p < 0.001 by two tailed unpaired t test. Multi-comparisons were corrected using FDR. The boxes represent the median and IQR, with the whiskers representing the data range (1.5 × IQR). Immune cell infiltration levels were predicted by deconvolution using the CIBERSORT algorithm. Differential immune cells with significance were marked in red color.

Functional enrichment analysis of the 455 candidate genes revealed significant involvement in critical biological processes. GO analysis indicated enrichment in the regulation of membrane potential, muscle system processes, myofibril and extracellular matrix organization (**Figure 2A**). KEGG pathway analysis highlighted focal adhesion, calcium signaling, and cGMP-PKG signaling pathways (**Figure 2B**), while Reactome analysis pointed to muscle contraction and extracellular matrix organization (**Figure 2C**). These findings suggested that the shared transcriptomic signature is predominantly associated with cell-matrix interactions and signaling pathways that regulate cellular contractility and ion homeostasis. This suggests that the comorbidity mechanism of AD and PCa may be related to inflammatory fibrosis in the central nervous system and epithelial mesenchymal transition (EMT) in the tumor, with stromal cells and components likely playing a concealed role in this process. The protein-protein interaction (PPI) network further visualized the connectivity among these key genes (**Supplementary Figure 2A**), indicating that multiple candidates may function in coordinated protein complexes.

Given that PCa is a typical immune cold tumor (32) and that inflammation and immune heterogeneity play important pathogenic roles in the pathogenesis of AD (33), we attempted to explore the common immune-pattern among them, by evaluating levels of general immune cell infiltration. We then performed deconvolution analysis on transcriptomic data of AD and PCa using the CIBERSORT algorithm to calculate and predict the infiltration levels of immune cells in disease groups versus normal control groups. Comparative analysis of immune cell infiltration revealed distinct compositional differences between disease and normal groups in both AD and PCa datasets using the False Discovery Rate (FDR) correction. In AD, T regulatory cells (Tregs), type 2 macrophages, monocytes and activated mast cells were significantly altered compared to normal controls, whereas in PCa, multiple immune subsets, including memory B cells, activated CD4^+^ T memory cells, follicular helper T cells, Tregs, resting & activated NK cells, monocytes, type 2 macrophages and neutrophils, exhibited substantial changes in abundance (**Figures 2D, E**; **Supplementary Figures 2B, C**). Notably, some immune cells were observed to share infiltration patterns across the two diseases. T cell follicular helper cells and resting mast cells were less abundant in disease groups, whereas Tregs and type 2 macrophages (M2) were enriched in both AD and PCa disease samples (**Figures 2D, E**). The parallel changes in immune cell composition across two distinct diseases suggested a potential shared immune-inflammatory mechanism underlying both pathologies, in addition to disease-specific immune responses.

### Development and evaluation of an AD-PCa-gene signature risk score model

**Figure 3:**
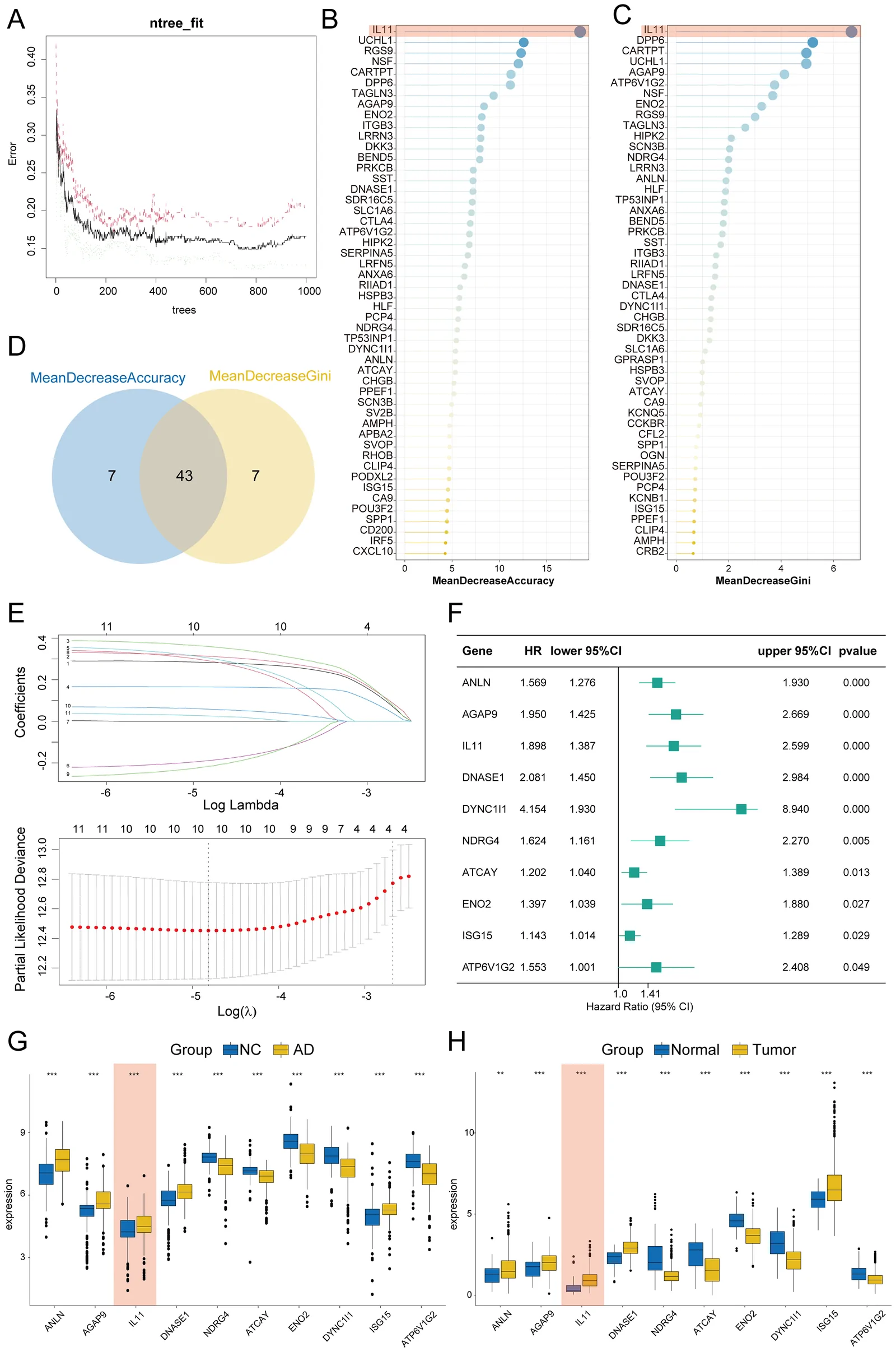
Machine learning screening of diagnostic and prognostic comorbidity genes for AD-PCa. **(A)** Biomarker screening based on the SVM-RFE algorithm in AD datasets. The lines represent the error rates as the number of decision trees increases: the black line typically denotes the Out-of-Bag (OOB) error, while other lines denote class-specific errors. **(B)** Lollipop plot showing the top 50 comorbidity genes ranked by importance based on Mean Decrease Accuracy (MDA) in the Random Forest (RF) model. The top ranked gene, IL11, is highlighted with a red rectangle. **(C)** Lollipop plot showing the top 50 comorbidity genes ranked by importance based on Mean Decrease Gini (MDG) in the Random Forest (RF) model. The top ranked gene, IL11, is highlighted with a red rectangle. **(D)** Venn diagram showing that a total of 43 genes exhibited high importance after evaluation by both MDA and MDG. **(E)** Biomarker screening based on the Lasso regression algorithm in AD datasets. The curve’s lowest point corresponded to the optimal gene count. Each curve in LASSO coefficient diagram represented one specific gene. The lines depict the coefficient profiles of the individual candidate genes as a function of the penalization parameter (Log Lambda). **(F)** Forest plot showing the prognostic value of 10 candidate comorbidity genes, ANLN, AGAP9, IL11, DNASE1, DYNC1I1, NDRG4, SPP1, ENO2, ISG15 and ATP6V1G2, selected by LASSO regression for progression-free interval (PFI) in prostate cancer, as evaluated by a multivariate Cox regression model. **(G)** Box plots comparing the expression levels of candidate comorbidity genes between the AD group and healthy control group samples. ***p < 0.001 by two tailed unpaired t test. Multi-comparisons were corrected using FDR. The boxes represent the median and IQR, with the whiskers representing the data range (1.5 × IQR). **(H)** Box plots comparing the expression levels of candidate comorbidity genes between the PCa group and normal group samples. **p < 0.01, ***p < 0.001 by two tailed unpaired t test. Multi-comparisons were corrected using FDR. The boxes represent the median and IQR, with the whiskers representing the data range (1.5 × IQR).

To refine the candidate gene pool into a clinically applicable model, we applied a two-tier machine learning pipeline. First, random forest (RF) analysis performed on the AD cohort identified 43 top ranked genes based on variable importance metrics, including MDA and MDG (**Figures 3A–D**). Notably, during the construction of this transcriptome based high throughput machine learning model, we found that interleukin 11 (IL11) exhibited the highest contribution in the RF model (**Figures 3B, C**). These candidate genes were then subjected to LASSO Cox regression in the TCGA-PRAD cohort to prevent overfitting. The optimal penalty parameter (λ) yielded a parsimonious signature consisting of ten genes: NDRG4, ISG15, IL11, ENO2, DYNC1I1, DNASE1, ATP6V1G2, ATCAY, ANLN, and AGAP9 (**Figure 3E**). To determine whether potential pathogenic genes for AD also have prognostic implications for PCa progression, we further incorporated these ten candidate genes into a multivariate Cox model for prostate cancer survival analysis to evaluate their diagnostic value for PCa prognosis (**Figure 3F**). The results further showed that all ten AD-PCa related genes were independent risk factors for PCa prognosis. Thus, through the machine learning screening pipeline, we effectively removed the noise introduced by high-throughput omics and ultimately retained ten diagnostic genes of AD and PCa from the 455 differential genes of dual diseases.

At the transcriptional level, these ten genes were consistently observed abnormally expressed in both PCa (tumor vs. normal, FDR < 0.05) (**Figure 3G**) and AD (disease vs. control, FDR < 0.05), in which IL11 was significantly expressed higher in the disease groups (**Figure 3H**). Meanwhile, we noticed that in both AD and PCa, these ten dual disease related genes were clustered into two distinct expression modules based on their expression patterns: IL11, ANLN, AGAP9, DNASE1, and ISG15 were upregulated in disease groups and showed a positive correlation trend among each other. In contrast, ATCAY, ENO2, NDRG4, DYNC1I1, and ATP6V1G2 were downregulated in disease samples and also exhibited significant co expression among themselves (**Supplementary Figures 2D, E**). By plotting ROC curves to evaluate the diagnostic efficacy of the single gene expression levels of these ten genes, it was found that NDRG4, ENO2, IL11, and DNASE1 had the highest diagnostic efficacy for AD, with all AUC values greater than 0.8 (**Supplementary Figure 2F**).

**Figure 4:**
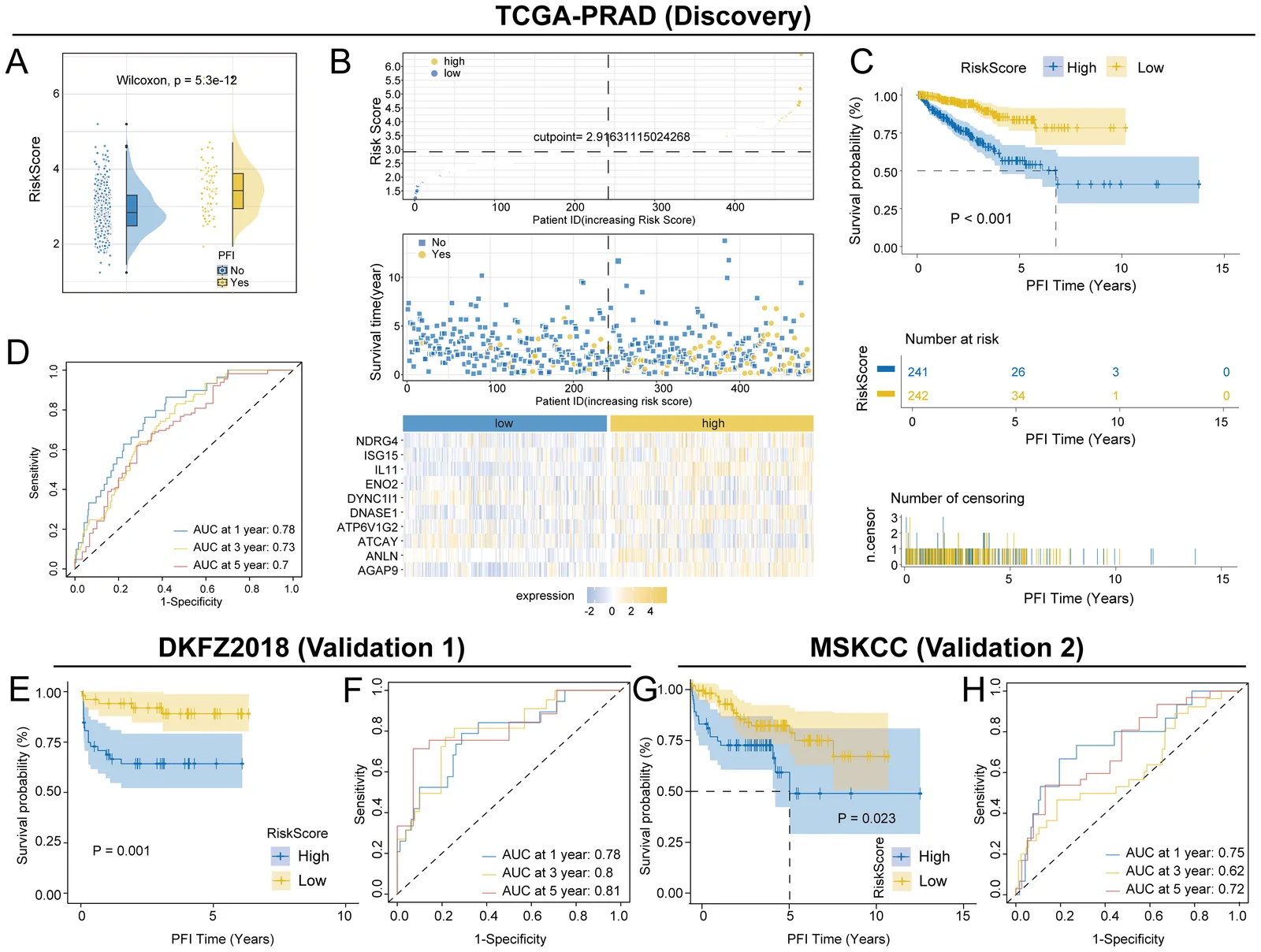
Construction of a comorbidity gene risk score signature model and validation of its diagnostic efficacy. **(A)** Comparison of risk scores between PCa samples with progression and without progression in the TCGA-PRAD cohort. **(B)** Combined scatter and heatmap plots showing the trend of cancer progression risk in TCGA-PRAD samples with increasing risk scores and upregulation of single gene expression. **(C)** Kaplan-Meier (K-M) curves comparing PFI between high risk and low risk score PCa samples in the TCGA-PRAD cohort. **(D)** ROC curves evaluating the diagnostic efficacy of the comorbidity gene risk score signature model for 1-, 3-, and 5-year PFI of PCa in the TCGA-PRAD cohort. **(E)** Validation of the prognostic value of the comorbidity gene risk score signature model for PCa PFI using K-M curves in DKFZ2018 cohort. **(F)** Validation of the diagnostic efficacy of the comorbidity gene risk score signature model for PCa PFI using ROC curves in DKFZ2018 cohort. **(G)** Validation of the prognostic value of the comorbidity gene risk score signature model for PCa PFI using K-M curves MSKCC cohort. **(H)** Validation of the diagnostic efficacy of the comorbidity gene risk score signature model for PCa PFI using ROC curves in MSKCC cohort.

Next, we constructed a gene risk score model by integrating expression levels of the10 AD-PCa genes, and assessed its cancer-prognostic value across multiple clinical cohorts of PCa. In TCGA-PRAD cohort, we found that individuals who experienced PCa progression during the follow-up period had significantly higher risk scores (Wilcoxon P = 5.3 × 10^-^¹²) (**Figure 4A**). For the overall PCa population, the risk of tumor progression increases with higher risk scores (**Figure 4B**); K-M analysis showed that PCa patients with higher risk scores had shorter progression free survival (log-rank P < 0.001) (**Figure 4C**). Moreover, a nomogram integrating the risk score was established to predict individual patient survival probabilities. The risk score demonstrated robust predictive accuracy, with AUC values of 0.78, 0.73, and 0.70 for 1-, 3-, and 5-year PFI survival, respectively (**Figure 4D**).

The model’s robustness was rigorously validated in two external independent cohorts, as well. In the DKFZ2018 cohort, the high-risk group showed significantly worse survival (log-rank P = 0.001) (**Figure 4E**), with AUCs exceeding 0.8 for 3- and 5-year prognostic prediction for PFI (**Figure 4F**). Similarly, in the MSKCC cohort, the signature successfully stratified patient prognosis (log-rank P = 0.023) (**Figure 4G**) and showed effective prediction of prognosis (**Figure 4H**). These independent validations confirmed the applicability of this signature across different patient populations and microarray-based expression platforms.

### IL11 relates to progression and low immunogenicity of PCa

Among the ten signature genes, IL11 was identified as a top candidate in the Random Forest analysis and exhibited the highest regression coefficient in the LASSO model. We therefore focused on dissecting its specific role. Pan-cancer analysis revealed that IL11 is significantly upregulated in tumor tissues compared to adjacent normal tissues across multiple cancer types, including PCa (**Supplementary Figure 3A**). This widespread upregulation suggested that IL11 may play a general oncogenic role rather than being restricted to prostate cancer. High IL11 expression was positively correlated with the infiltration of mast cells, macrophages, and neutrophils (**Supplementary Figure 3B**), indicating a potential link between IL11 and the recruitment or modulation of myeloid lineage cells in the tumor microenvironment. Furthermore, high IL11 expression was associated with higher genomic instability in PCa samples, including elevated tumor mutation burden (TMB) and specific somatic mutations (**Supplementary Figure 3C**). Based on this, we again performed deconvolution analysis on the transcriptomic data to calculate and compare whether there were differences in immune cell infiltration between PCa tissues with high and low IL11 expression (**Supplementary Figures 3D–F**). It turned out that in the IL11-high group, effector lymphocytes with antitumor functions, such as plasma cells and CD8+ T cells, showed lower abundance, whereas immunosuppressive Tregs and M2 macrophages were relatively higher (**Supplementary Figure 3G**). This suggests that high expression of IL11 in PCa may play an immunosuppressive regulatory role.

**Figure 5:**
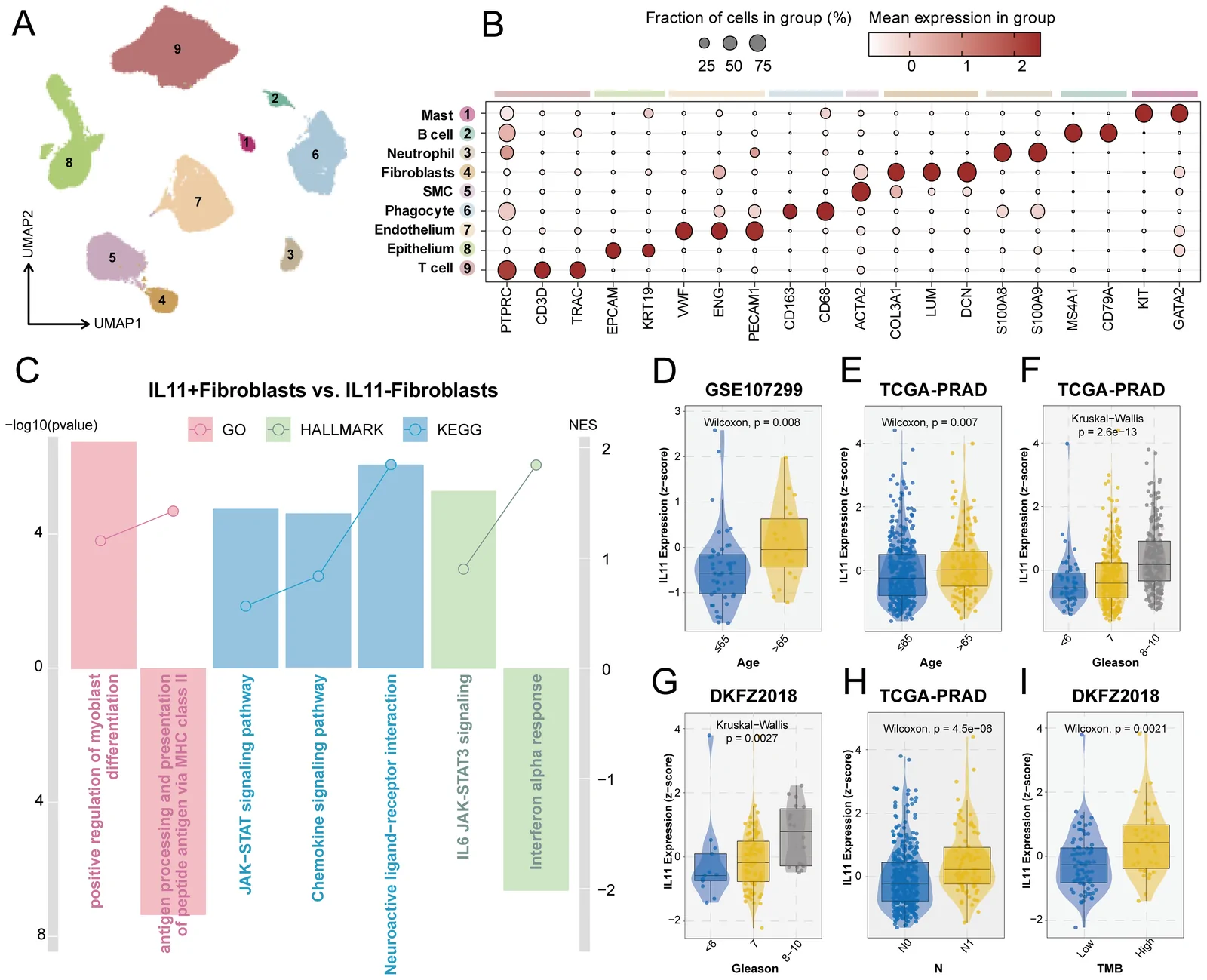
Exploration of the function of IL11 in PCa progression. **(A)** UMAP showing dimensionality reduction clustering of major cell populations based on human PCa single-cell transcriptome data. **(B)** Bubble plot showing the expression of marker genes used for cell annotation. **(C)** Bar plot showing comparison of GSEA differential pathways between IL11+_Fibro cells and IL11-_Fibro cells based on GO, KEGG, and HALLMARK databases. **(D)** Box plots showing significantly higher IL11 expression in elder PCa samples over 65 years old in GSE107299 dataset. **(E)** Box plots showing significantly higher IL11 expression in elder PCa samples over 65 years old in TCGA-PRAD dataset. **(F)** Box plots showing significantly higher IL11 expression in PCa samples with advanced Gleason grades in TCGA-PRAD dataset. **(G)** Box plots showing significantly higher IL11 expression in PCa samples with advanced Gleason grades in DKFZ2018 dataset. **(H)** Box plots showing significantly higher IL11 expression in PCa samples with N1 stage in TCGA-PRAD dataset. **(I)** Box plots showing significantly higher IL11 expression in PCa samples with higher TMB levels in TCGA-PRAD dataset.

Since IL11 is an important immune cytokine involved in inflammatory fibrosis and matrix remodeling (34–37), we further localized the source of IL11 in PCa using single cell transcriptomic data from 13 human PCa tissues (GSE141445) (38). Interestingly, after identifying and annotating the major cell populations (**Figures 5A, B**), we found that IL11 was specifically expressed in fibroblasts (highly expressed marker genes FAP, PDGFRA, PDGFRB, and ACTA2) (**Supplementary Figures 4A–D**), whereas its receptor, encoded by the gene IL11RA, was broadly distributed across all major cell groups (**Supplementary Figure 4E**). This suggested that cancer associated fibroblasts (CAFs) may participate in the regulation of the PCa immune microenvironment through the synthesis and secretion of IL11. We therefore defined fibroblasts with high and low expression of IL11 as IL11 positive fibroblasts (Fibro_IL11+) and IL11 negative fibroblasts (Fibro_IL11-), respectively, to identify the potential functions of CAFs with high IL11 synthesis and secretion activity in the immune microenvironment. Differential pathway enrichment analysis showed that in Fibro_IL11+ cells, pathways related to terminal mesenchymal myofibroblast differentiation, IL6 associated JAK STAT signaling, cytokine signaling, and excitatory neurotransmitter signaling were significantly activated, whereas MHC class II antigen presentation and interferon alpha response pathways were significantly suppressed (**Figure 5C**). This indicated that high expression of IL11 promotes EMT, stemness, and the production of immunosuppressive secretory signals, thereby exerting a protumorigenic function. Meanwhile, CellChat-based cell communication analysis revealed that Fibro_IL11^+^ cells, which exhibit high IL11 expression, engage in complex communication networks with multiple cell types in the microenvironment (**Supplementary Figure 4F**). Clinically, elevated IL11 levels correlated with older ages, advanced Gleason scores, N stages and TMB levels in multiple PCa cohorts (**Figures 5D–I**). Survival analysis in TCGA-PRAD demonstrated that high IL11 expression is a strong predictor of poor disease-free survival (DFS), disease-specific survival (DSS), and PFI (**Supplementary Figures 4G–J**). This prognostic significance of relapse-free survival (RFS) was also validated in four external cohorts (**Supplementary Figures 4K–N****),** including DKFZ2018, GSE21034, GSE107299 and GSE116918. Collectively, these results established IL11 as a robust biomarker for PCa progression and suppressive immune microenvironment.

Previous literatures have reported that IL11/IL11RA ligand-receptor pair constitutes a well-established, mature secreted inflammatory cytokine signaling axis that participates in diverse matrix-remodeling biological events within the microenvironment (37, 39). As IL11 binds to its specific receptor IL11RA in the microenvironment to trigger downstream pathways to mediate its biological effects (40), we further investigated whether IL11RA is associated with disease progression in PCa (**Supplementary Figure 5A**). We observed a significant positive correlation between IL11 and IL11RA mRNA expression in PCa tissue samples from TCGA-PRAD dataset (**Supplementary Figure 5B**). Notably, IL11RA expression was significantly higher in SPOP-mutated PCa samples (**Supplementary Figure 5C**), indicating that SPOP mutations may enhance the responsiveness of prostate cancer cells to IL11 signaling. Immunohistochemistry (IHC) data of five PCa tissues from the Human Protein Atlas (HPA) confirmed that IL11RA protein levels are elevated in high-grade PCa tissues (**Supplementary Figure 5D**), supporting the transcriptomic findings at the protein level.

Consistent with IL11, higher IL11RA expression in PCa also implied poorer DFS, PFI and RFS for the patients across TCGA-PRAD, GSE21034 and GSE116918 datasets (**Supplementary Figures 5E–H**). Furthermore, evaluation using CIBERSORT algorithm provides an exploratory landscape of the complex, albeit heterogeneous, associations between IL11RA expression and immune infiltration across different platforms. It was found that IL11RA expression was also positively associated with immunosuppressive immune cells, particularly M2 macrophages and regulatory T cells (Tregs), in bulk tumor tissues across multiple cohorts (**Supplementary Figure 5I**).

### Functional validation of the pro-disease effects of IL11 in PCa and AD

To explore the biological function of IL11, we performed functional enrichment and wet-lab experiments. Gene set enrichment analysis (GSEA) showed that high expression of IL11 in AD led to the activation of multiple biological events, including various neurodegenerative disease related processes including AD, stress response like reactive oxygen and unfolded protein, as well as metabolism related glycolysis and androgen response (**Supplementary Figure 6A**), while upregulated IL11 in PCa promoted anti-inflammatory and pro-cancer pathways such as EMT, androgenesis, IL2 signaling, and hypoxia (**Supplementary Figure 6B**). Differential analysis highlighted pathways related to cytokine interactions and metabolic processes (**Supplementary Figures 6C, D**). These results suggested that IL11 may exert context-dependent effects, with a focus on metabolic regulation in AD and on invasive phenotypes in PCa.

**Figure 6:**
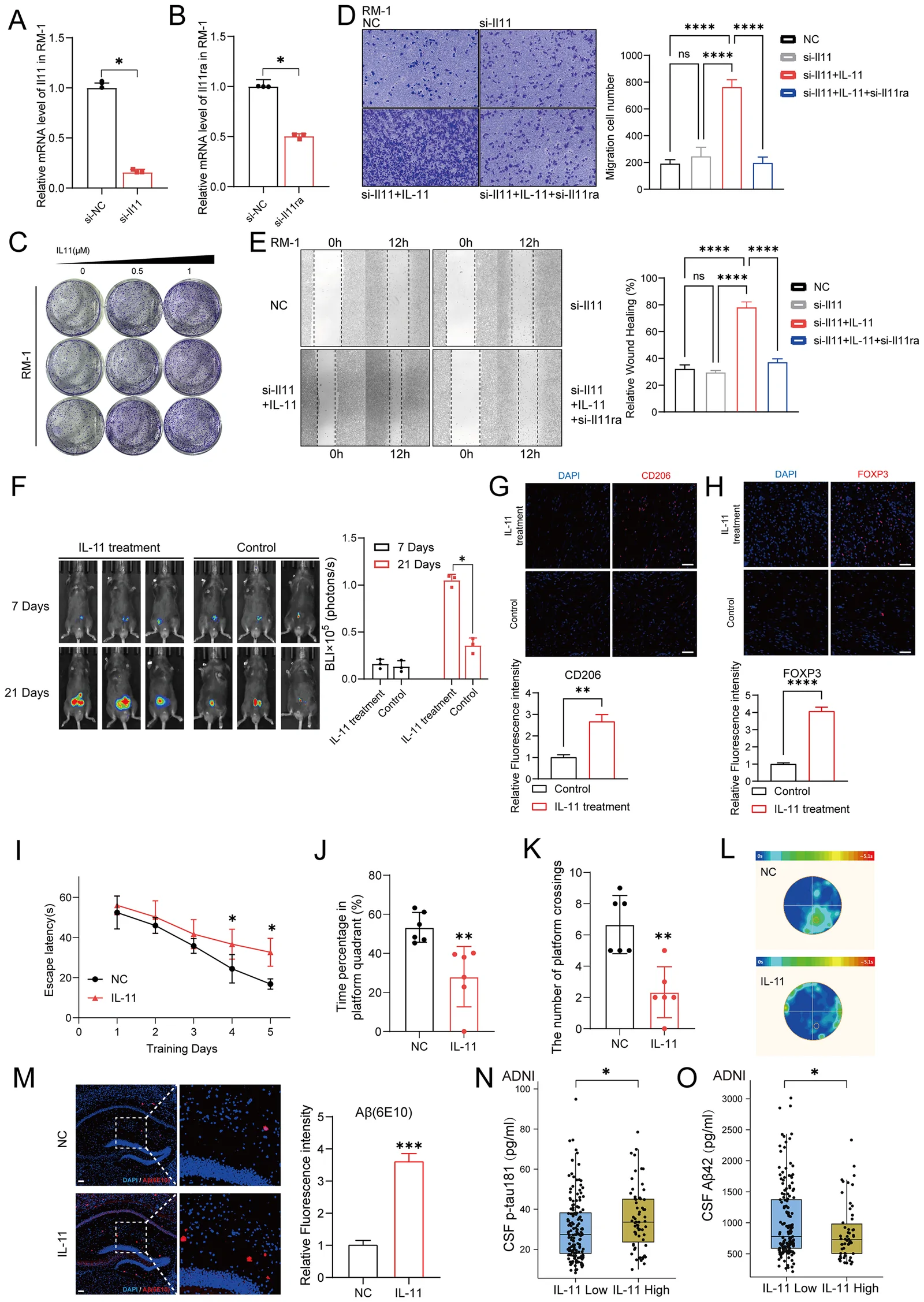
Functional prediction and validation of IL11 in the development of PCa and AD. **(A)** Bar graph showing the knockdown efficiency of IL11 in RM-1 cells as determined by qRT-PCR. *p < 0.05 by two tailed unpaired t test, compared to si-NC group. **(B)** Bar graph showing the knockdown efficiency of IL11RA in RM-1 cells as determined by qRT-PCR. *p < 0.05 by two tailed unpaired t test, compared to si-NC group. **(C)** Colony formation assay examining the effect of IL11 intervention on the growth of the mouse PCa cell line RM-1. **(D)** Wound healing assay examining the effect of IL11 intervention on the invasiveness of RM-1 cells. Bar plot showing the results of quantitative comparison between groups. ****p < 0.0001 by two tailed unpaired t test. **(E)** Transwell assay examining the effect of IL11 intervention on the migration ability of RM-1 cells. ****p < 0.0001 by two tailed unpaired t test. **(F)** *In vivo* bioluminescence imaging, and quantification in bar plots of orthotopic prostate tumor growth in mice between two groups. *p < 0.05 by two tailed unpaired t test. BLI, bioluminescence intensity. **(G)** IF staining and quantitative analyses of CD206 (red) abundance in orthotopic prostate tumors of mice across IL11-treated and control groups. Scale bar = 50 μm. **p < 0.01 by two tailed unpaired t test. **(H)** IF staining and quantitative analyses of FOXP3 (red) abundance in orthotopic prostate tumors of mice across IL11-treated and control groups. Scale bar = 50 μm. ****p < 0.0001 by two tailed unpaired t test. **(I)** Escape latency of IL11-treated mice and the controls for 5 consecutive days in the MWM test. *p < 0.05 by two tailed unpaired t test, compared to NC group. **(J)** Percentage of time spent in the platform quadrant in the spatial probe trial of the MWM test. **p < 0.01 by two tailed unpaired t test, compared to NC group. **(K)** The number of platform crossings in the spatial probe trial of the MWM test. **p < 0.01 by two tailed unpaired t test, compared to NC group. **(L)** Representative heat maps of swimming paths in the spatial probe trial of the MWM test. **(M)** IF staining and quantitative analyses of Aβ (6E10, red) in the hippocampus of IL11-treated and control mice. Scale bar = 100 μm. ***p < 0.001 by two tailed unpaired t test. **(N)** Boxplots of CSF p-tau 181 levels in high-IL-11 (top 25%) and low-IL-11 (bottom 75%) groups in ADNI cohort. Groups were compared by Welch’s t-test. The boxes represent the median and interquartile range (IQR), with the whiskers representing the data range (1.5 × IQR). **(O)** Boxplots of CSF Aβ42 levels in high-IL-11 (top 25%) and low-IL-11 (bottom 75%) groups in ADNI cohort. Groups were compared by Welch’s t-test. The boxes represent the median and interquartile range (IQR), with the whiskers representing the data range (1.5 × IQR).

To confirm this conclusion, we first used the human PCa cell line DU145 to evaluate the pro-cancer effect of IL11. *In vitro* colony formation (**Supplementary Figure 6E**), wound healing assay (**Supplementary Figure 6F**), and Transwell migration assay (**Supplementary Figure 6G**) all demonstrated that DU145 cells treated with IL11 exhibited enhanced malignancy and higher invasiveness. Because DU145 cells constitutively secrete high levels of IL11 that may act in a paracrine manner, we next sought to validate these findings while excluding confounding by endogenous IL11 production. We therefore turned to the murine RM-1 PCa cell line and silenced endogenous Il11 and Il11ra using small interfering RNA (si-Il11 & si-Il11ra) (**Figures 6A, B**). On this IL11-depleted background, exogenous IL11 treatment again significantly enhanced the malignant behaviors of RM-1 cells, including clonogenic capacity in the colony formation assay, gap closure in the wound healing assay, and migratory ability in the Transwell assay (**Figures 6C–E**), whereas si-Il11 knockdown alone reduced these phenotypes. Conversely, knockdown of Il11ra in RM-1 cells abrogated the promoting effects of exogenous IL11 on the malignant behaviors of PCa cells (**Figures 6C–E**). These results closely paralleled those obtained in DU145 cells and confirmed that the pro-tumoral effect of IL11 is independent of endogenous cytokine production.

Further using the RM-1-C57BL/6 mouse system, we first investigated the effect of *in vivo* IL11 treatment on the growth of mouse xenograft tumors using an orthotopic tumor model. Fluorescence *in vivo* imaging of orthotopic PCa xenografts revealed that tumor growth in the IL11-treated group was significantly stronger than that in the control group (**Figure 6F**). The mice subcutaneous tumor transplantation model of PCa also showed that mice receiving intraperitoneal injection of IL11 exhibited faster tumor growth and greater final tumor weight (**Supplementary Figure 6H**). Taken together, the *in vitro* and *in vivo* results provide converging evidence that IL11 drives PCa progression and tumor growth, supporting its candidacy as a functional driver in the shared pathogenesis of PCa. To investigate the impact of IL11 on immune cell infiltration in PCa, we examined FOXP3^+^ Tregs and CD206^+^ M2 macrophages in orthotopic tumors by IF. Both markers exhibited markedly higher expression in the IL11-treated group compared with controls (**Figures 6G, H**).

On the other hand, we also conducted independent mouse behavioral experiments to verify the effect of IL11 on cognitive function. During the 5-day training phase of the MWM test, the mice in the IL11 treatment group displayed prolonged escape latencies compared with that of the control group, especially on the 4th day and 5th day (**Figure 6I**). In the spatial probe test, IL11-treated mice also performed worse than the controls, as reflected by the lower percentage of time spent in the target quadrant (**Figure 6J**) and the fewer number of platform crossings (**Figure 6K**). The heat maps in the spatial probe trial showed that the mice in the control group swam around the platform, while the mice in the IL11-treated group swam around the areas away from the platform (**Figure 6L**). To determine whether these cognitive deficits were accompanied by AD-like neuropathological changes, we examined hippocampal Aβ deposition via immunofluorescence. Immunofluorescence results demonstrated a significant increase in hippocampal Aβ expression in IL11-treated mice relative to control mice (**Figure 6M**). Collectively, these behavioral and pathological findings indicate that IL11 treatment not only induces significant cognitive impairment but also promotes hippocampal Aβ accumulation.

To directly bridge the gap between peripheral IL-11 and central AD pathology in humans, we performed a comprehensive cross-sectional analysis using the ADNI database, including 215 participants with concurrent measurements of serum IL-11 and CSF core AD biomarkers (Aβ42 and p-tau181). Serum IL-11 levels were significantly elevated in patients with AD compared with HCs in ADNI cohort (**Supplementary Figure 6I**). Serum IL-11 levels were negatively correlated with cognitive function as assessed by the MMSE score, suggesting that higher IL-11 was associated with cognitive decline (**Supplementary Figure 6J**). When subjects were stratified by quartiles of serum IL-11 levels, those in the highest quartile exhibited significantly higher CSF p-tau181 levels (**Figure 6N**) and significantly lower CSF Aβ42 levels relative to those in the lowest quartile (**Figure 6O**), indicating accumulation of Aβ and phosphorylated tau in the brain.

To sum up, through an integrated bioinformatics analysis along with machine learning methods, this study identified and verified shared genes and signaling pathways between PCa and AD, with IL11 being the most prominent functional candidate, aiming to identify novel diagnostic biomarkers and therapeutic targets preclinically.

## Discussion

The clinical and scientific challenge of managing two major aging-related diseases, PCa and AD, has long been complicated by fragmented views that treat them as unrelated entities. Although epidemiological studies have sometimes noted an inverse statistical association, growing evidence suggests that the same aging related inflammatory and senescent pathways can predispose individuals to both conditions, especially when considering the long-term effects of cancer therapies such as ADT, which increases AD risk (13, 14, 41, 42). To move beyond isolated disease paradigms, we integrated multi cohort transcriptomic data with a systematic machine learning pipeline. We initially utilized the Random Forest (RF) algorithm to screen for robust candidate genes by taking the intersection of the top 50 genes based on two key metrics: Mean Decrease Accuracy (MDA) and Mean Decrease Gini (MDG). MDA evaluates a gene’s contribution to the model’s predictive accuracy, while MDG measures its impact on node purity and classification capability. However, high importance in the RF model does not necessarily equate to prognostic relevance. Therefore, rather than carrying all intersected genes forward, we subsequently applied Cox and LASSO regression analyses to rigorously evaluate their prognostic efficacy. Only genes demonstrating statistical significance in these survival-based analyses were retained for subsequent model construction. This sequential filtration strategy was deliberately designed to eliminate redundant variables, minimize the risk of overfitting, and ensure the final model is both highly efficient and clinically applicable. This approach allowed us to identify a ten gene shared signature, with IL11 as the top candidate. Using *in vitro* functional assays, an immunocompetent xenograft model, mouse behavioral tests, and brain pathological examination, we demonstrated that IL11 was related to PCa progression and cognitive impairment. Collectively, our findings position IL11 as a convergent molecular hub that may correlate with both diseases through overlapping yet context dependent pathways, supporting the notion of a shared pathological continuum rather than a strictly inverse relationship.

Prior attempts to untangle the PCa-AD connection have largely relied on observational or MR designs, yielding inconsistent conclusions. Some MR studies have suggested a causal effect of AD on lowering PCa risk, while others found no significant effect in the opposite direction (11, 12). These discrepancies likely reflect the complexity of aging related biology and the fact that traditional MR captures lifelong genetic predisposition but not the dynamic, treatment induced changes occurring in elderly patients. Moreover, most previous investigations focused on disease specific mechanisms, such as amyloid cascade in AD or androgen receptor signaling in PCa, without systematically searching for overlapping molecular drivers. In contrast, our study employed a unified analytical framework that combined differential expression, WGCNA, RF, and LASSO Cox regression. To systematically identify key pathogenic genes shared between PCa and AD, we first performed an unbiased genome-wide differential expression analysis to delineate the full repertoire of genes commonly dysregulated in both diseases (**Figures 1**, **2**). This initial screening step yielded a broad gene set and its enriched pathways, without any presupposition regarding which gene might be the most critical. Subsequently, to prioritize the most biologically relevant candidates from this pool, we applied a series of machine-learning algorithms and regression models for feature selection. Through this sequential narrowing-down process, IL11 emerged as the top-ranked risk factor strongly associated with disease development and progression (**Figure 3**). Therefore, IL11 is not individually highlighted at the beginning, as these panels depict the initial, unfiltered screening results; it becomes the central focus only after the computational prioritization, which is the basis for the subsequent functional investigations.

The addition of single cell transcriptomics and multi algorithm immune deconvolution (CIBERSORT, ESTIMATE, TIMER, etc.) provided a detailed view of IL11’s cellular origin and its impact on the immune microenvironment. To our knowledge, this is the first study to experimentally validate IL11 as a functional comorbidity factor linking PCa and AD using an integrated bioinformatics to bench pipeline, thereby offering a new perspective on the shared pathogenesis of these two devastating disorders. A recent dose response meta-analysis further confirmed that ADT is associated with a significantly elevated dementia risk, highlighting the need for cross disease investigations (43).

IL11, a member of the IL-6 cytokine family, has traditionally been viewed as a hematopoietic factor, but recent work has redefined it as a pathological mediator of fibrosis and inflammation (35, 44–46). In PCa, IL11/IL11RA signaling has been shown to promote chemoresistance via the JAK/STAT axis (4, 47). Our single cell analysis of human PCa tissues revealed that IL11 is predominantly expressed by a subset of CAFs, whereas its receptor IL11RA is broadly distributed across multiple cell types. This expression pattern suggests that CAFs may secrete IL11 to orchestrate a paracrine network that drives EMT and recruits immunosuppressive cells. Indeed, we observed that high IL11 expression correlated with increased infiltration of M2 macrophages and Tregs, as well as elevated tumor mutation burden. In the brain, IL11 expression is normally low but becomes upregulated under neuroinflammatory conditions (48, 49). Clinical studies have also confirmed that CSF IL-11 levels are significantly elevated in patients with AD (49). Our GSEA results showed that high IL11 expression in AD is associated with stress responses (reactive oxygen species, unfolded protein), metabolic reprogramming (glycolysis), and multiple neurodegenerative pathways. In this study, data from both animal experiments and human cohort studies suggest that IL-11 may be involved in the core pathological process of AD and is closely associated with cognitive impairment. Recent studies have linked IL-11 to microglial NLRP3 inflammasome activation and NF-κB signaling, which may be one of the mechanisms underlying the exacerbation of AD pathology (50). Based on these findings, we propose a convergent model: in both PCa and AD, chronic inflammation, whether arising from the tumor microenvironment or the aging brain, upregulates IL11, which then activates overlapping signaling cascades (JAK/STAT, NF-κB, MAPK/ERK) (51). In PCa, this promotes EMT (52), immune evasion (53), and tumor growth (52, 53); in AD, it exacerbates neuroinflammation (49, 50), synaptic dysfunction (54), and cognitive decline (50, 54). The divergent outcomes likely reflect the distinct cellular contexts and tissue architectures in which IL11 operates.

Several limitations of this study should be acknowledged. First, transcriptomic data alone cannot establish causality; future Mendelian randomization studies using GWAS summary statistics are needed to determine whether IL11 is a causal mediator or merely a correlate of shared aging pathways. Second, our xenograft models do not fully recapitulate the native PCa microenvironment; and genetically engineered models would be more informative. In particular, while our single-cell transcriptomic data identified CAFs as a major source of IL11 in the tumor microenvironment, the relative contribution of CAF-derived versus tumor cell-derived IL11 to the observed phenotypes remains to be fully dissected by further co-culture system or tissue validation. For AD, IL11 modified transgenic models with longitudinal cognitive assessment are warranted. Third, the downstream signaling crosstalk between IL11 and androgen receptor remains unclear. Also, we recognize that the absence of publicly available AD datasets with longitudinal survival data precluded reverse validation of PCa-derived risk genes for AD prognosis. As most AD transcriptomic resources are cross-sectional, we could not perform this reciprocal analysis. Nonetheless, our unbiased screening workflow and the robust differential expression of IL11 in both diseases, supported by independent functional evidence, underpin the validity of our findings, pending future studies with properly annotated AD survival cohorts. Fourth, while our findings establish a correlative and functional link between IL11 and AD, the precise mechanisms by which IL11 communicates with the central nervous system, including potential BBB penetration, activation of glial cells, or engagement of peripheral to central inflammatory signaling warrant further investigation. Finally, although an IL11Rα targeting agent (BMTP-11) has shown early promise in metastatic PCa (55), we did not test IL11 pathway inhibition in AD. Given that neutralizing anti-IL11 antibodies are effective in fibrosis models (44, 45), evaluating such interventions in both diseases is a logical next step.

In conclusion, this study identifies IL11 as a shared risk factor that relates to PCa progression and cognitive decline in AD through converging molecular pathways, highlighting the IL11/IL11RA axis as a promising therapeutic target for PCa and AD.

## Data Availability

The original contributions presented in the study are included in the article/**Supplementary Material**. Further inquiries can be directed to the corresponding author.

## Supplementary material

Supplementary Figure 1 Processing of human AD transcriptome data and WGCNA strategy, related to **Figure 1**. **(A)** Principal component analysis (PCA) showing the variation of principal components between AD and NC groups before and after batch effect removal across three AD RNA-seq datasets. **(B)** PCA showing the variation of principal components between three AD RNA-seq datasets before and after batch effect removal. **(C)** The selection of soft threshold in AD datasets. **(D)** The selection of soft threshold in TCGA-PRAD. **(E)** Heatmap showing the clustering of genes into corresponding modules in WGCNA of the AD datasets. **(F)** Heatmap showing the clustering of genes into corresponding modules in WGCNA of TCGA-PRAD. **(G)** Heatmap and dendrogram showing the final characteristic modules and branches from WGCNA of the AD dataset. **(H)** Heatmap and dendrogram showing the final characteristic modules and branches from WGCNA of TCGA-PRAD. **(I)** Escape latency of IL11-treated mice and the controls for 5 consecutive days in the MWM test. *p < 0.05 by two tailed unpaired t test, compared to NC group. **(J)** Percentage of time spent in the platform quadrant in the spatial probe trial of the MWM test. **p < 0.01 by two tailed unpaired t test, compared to NC group. **(K)** The number of platform crossings in the spatial probe trial of the MWM test. **p < 0.01 by two tailed unpaired t test, compared to NC group. **(L)** Representative heat maps of swimming paths in the spatial probe trial of the MWM test.

Supplementary Figure 2 Functional prediction of the dual-disease-associated candidate genes, related to **Figures 2**, **3**. **(A)** Protein-protein interaction (PPI) network analysis of encoding comorbidity genes between AD and PCa. Proteins that directly or indirectly interact with IL11 and co-participate in biological pathways are highlighted in red. **(B)** Heatmap showing an overview of the infiltration and distribution of 22 immune cell types in AD and healthy control samples. **(C)** Heatmap showing an overview of the infiltration and distribution of 22 immune cell types in PCa and normal samples. Immune cell infiltration levels were predicted by deconvolution using the CIBERSORT algorithm. **(D)** Heatmap showing the expression correlations among candidate comorbidity genes in AD samples. Spearman’s test was used to assess the linear correlation of expression levels between each pair of genes. Spearman’s correlation analyses were performed, and P-values were adjusted for multiple comparisons using the Holm method. Adjusted P-values are presented in the figure. *FDR < 0.05, **FDR < 0.01, ***FDR < 0.001. **(E)** Heatmap showing the expression correlations among candidate comorbidity genes in PCa samples. Spearman’s test was used to assess the linear correlation of expression levels between each pair of genes. Spearman’s correlation analyses were performed, and P-values were adjusted for multiple comparisons using the Holm method. Adjusted P-values are presented in the figure. *FDR < 0.05, **FDR < 0.01, ***FDR < 0.001. **(F)** ROC curves evaluating the individual diagnostic efficacy of the 10 candidate comorbidity genes in AD.

Supplementary Figure 3 Analysis of immune cell correlation of IL11 in PCa. **(A)** Pan-cancer comparison of IL11 expression differences between tumor and adjacent normal tissues. *p < 0.05, **p < 0.01, ****p < 0.0001 by two tailed unpaired t test. **(B)** Heatmap showing the correlation of IL11 with 22 immune cell types across pan-cancer. Spearman’s test was used to assess the linear correlation. Spearman’s correlation analyses were performed, and P-values were adjusted for multiple comparisons using the Holm method. Adjusted P-values are presented in the figure. *FDR < 0.05, **FDR < 0.01, ***FDR < 0.001. **(C)** Heatmap comparing key driver genes and genome-wide mutations between IL11 high and low expression samples of PCa. *p < 0.05, **p < 0.01, ***p < 0.001. **(D)** Heatmap showing the correlations among 22 immune cell types in PCa samples with high IL11 expression. Immune cell abundances were predicted by deconvolution using the CIBERSORT algorithm. Spearman’s correlation analyses were performed, and P-values were adjusted for multiple comparisons using the Holm method. Adjusted P-values are presented in the figure. *FDR < 0.05, **FDR < 0.01, ***FDR < 0.001. **(E)** Heatmap showing the correlations among 22 immune cell types in PCa samples with low IL11 expression. Immune cell abundances were predicted by deconvolution using the CIBERSORT algorithm. Spearman’s correlation analyses were performed, and P-values were adjusted for multiple comparisons using the Holm method. Adjusted P-values are presented in the figure. *FDR < 0.05, **FDR < 0.01, ***FDR < 0.001. **(F)** Heatmap overviewing the differences in abundance of 22 immune cell types between PCa samples with high and low IL11 expression. Immune cell abundances were predicted by deconvolution using the CIBERSORT algorithm. **(G)** Box plots comparing the differences in abundance of 22 immune cell types between PCa samples with high and low IL11 expression. *p < 0.05, **p < 0.01, ***p < 0.001 by two tailed unpaired t test. Multi-comparisons were corrected using FDR. The boxes represent the median and IQR, with the whiskers representing the data range (1.5 × IQR).

Supplementary Figure 4 Single-cell characterization and clinical prognostic value of IL11 in human PCa, related to **Figure 5**. **(A)** Feature plot and violin plot showing expression of ACTA2 in all cell types. **(B)** Feature plot and violin plot showing expression of FAP in all cell types. **(C)** Feature plot and violin plot showing expression of PDGFRA in all cell types. **(D)** Feature plot and violin plot showing expression of PDGFRB in all cell types. **(E)** UMAP showing the expressions and distributions of IL11 and IL11RA in all celltypes of human PCa. **(F)** Cell-cell communication network mediated by IL11, based on CellChat analyses. **(G)** K-M curves evaluating the prognostic impact of IL11 expression of DFS in TCGA-PRAD cohort. Patients were stratified into high- and low-IL11 expression groups using the optimal cutoff value. **(H)** K-M curves evaluating the prognostic impact of IL11 expression of DSS in TCGA-PRAD cohort. Patients were stratified into high- and low-IL11 expression groups using the optimal cutoff value. **(I)** K-M curves evaluating the prognostic impact of IL11 expression of OS in TCGA-PRAD cohort. Patients were stratified into high- and low-IL11 expression groups using the optimal cutoff value. **(J)** K-M curves evaluating the prognostic impact of IL11 expression of PFS in TCGA-PRAD cohort. Patients were stratified into high- and low-IL11 expression groups using the optimal cutoff value. **(K)** K-M curves evaluating the prognostic impact of IL11 expression of RFS in DKFZ2018 cohort. Patients were stratified into high- and low-IL11 expression groups using the optimal cutoff value. **(L)** K-M curves evaluating the prognostic impact of IL11 expression of RFS in GSE21034 cohort. Patients were stratified into high- and low-IL11 expression groups using the optimal cutoff value. **(M)** K-M curves evaluating the prognostic impact of IL11 expression of RFS in GSE107299 cohort. Patients were stratified into high- and low-IL11 expression groups using the optimal cutoff value. **(N)** K-M curves evaluating the prognostic impact of IL11 expression of RFS in GSE116918 cohort. Patients were stratified into high- and low-IL11 expression groups using the optimal cutoff value.

Supplementary Figure 5 Preliminary exploration of the role of IL11-IL11RA in PCa progression and immunity. **(A)** Schematic diagram of the membrane binding interaction between IL11 and IL11RA. **(B)** Violin plot comparing the expression differences of IL11RA between PCa samples with high and low IL11 expression. **(C)** Violin plot comparing IL11RA expression differences between SPOP1 mutant and wild-type PCa samples. **(D)** Comparison of IL11R protein expression between high-grade and low-grade regions of five representative PCa samples based on the IHC data from Human Protein Atlas (HPA) database. **(E)** K-M curves assessing the impact of IL11RA expression levels on PCa prognosis for DFS in TCGA-PRAD cohort. **(F)** K-M curves assessing the impact of IL11RA expression levels on PCa prognosis for PFS in TCGA-PRAD cohort. **(G)** K-M curves assessing the impact of IL11RA expression levels on PCa prognosis for RFS in GSE21034 cohort. **(H)** K-M curves assessing the impact of IL11RA expression levels on PCa prognosis for RFS in GSE116918 cohort. **(I)** Heatmap showing the correlation between immune cell abundance and IL11RA expression levels across multiple PCa cohorts. Immune cell abundance levels were predicted by deconvolution using the ESTIMATE, MCPcounter, TIMER and xCell algorithms. Spearman’s test was used to assess the linear correlation.

Supplementary Figure 6 Molecular functions of IL11 in AD and PCa pathogenesis, related to **Figure 6**. **(A)** Bar plot showing the biological signaling pathways activated by IL11 in AD, analyzed with GSEA. **(B)** Bar plot showing the biological signaling pathways activated by IL11 in PCa, analyzed with GSEA. **(C)** Volcano plot showing the significantly enriched biological signaling pathways of IL11 related genes in AD based on GO and KEGG databases. **(D)** Volcano plot showing the significantly enriched biological signaling pathways of IL11 related genes in PCa based on GO and KEGG databases. **(E)** Colony formation assay examining the effect of IL11 intervention on the growth of the human PCa cell line DU145. **(F)** Wound healing assay examining the effect of IL11 intervention on the invasiveness of the human PCa cell line DU145. Bar plot showing the results of quantitative comparison between groups. **p < 0.01 by two tailed unpaired t test, compared to NC group. **(G)** Transwell assay examining the effect of IL11 intervention on the migration ability of the human PCa cell line DU145. ****p < 0.0001 by two tailed unpaired t test, compared to NC group. **(H)** Terminal images and growth curves of RM-1 subcutaneous tumors comparing the effect of IL11 on PCa tumor growth *in vivo*. Bar plot comparing final tumor weight between the IL11 treated group and the control group. **p < 0.01 by two tailed unpaired t test, compared to NC group. **(I)** Violin plots of serum IL-11 levels in HC, MCI, and AD groups in ADNI cohort. IL-11 abundance is shown as NPX (normalized protein expression) on a log_2_ scale. Data are mean ± SD. *p < 0.05 by One-way ANOVA with Tukey’s HSD post-hoc test was used for group comparisons. **(J)** Scatter plot displays the relationship between IL-11 and MMSE scores in ADNI cohort using multiple linear regression analysis, adjusting for age, sex, education, smoking, alcohol consumption. Standardized β is shown.
